# Effects of an Exercise Intervention Based on mHealth Technology on the Physical Health of Male University Students With Overweight and Obesity: Randomized Controlled Trial

**DOI:** 10.2196/69451

**Published:** 2025-07-31

**Authors:** Qinlong Zhang, Zhen Li, Lei Jiang, Yanan Gao, Tingjun Gong, Jian Li, Zhixiong Zhou

**Affiliations:** 1 Capital University of Physical Education and Sports, No.11, North Third Ring West Road, Haidian District, Beijing, 100191, China, 86 010 82099489; 2 School of Physical Education and Sport Science, Fujian Normal University, Fuzhou, China

**Keywords:** health promotion, physical activity, monitoring, dose-effect relationship, mobile phone

## Abstract

**Background:**

Obesity has become one of today’s global health challenges. According to the World Health Organization, in 2022, a total of 2.5 billion adults aged 18 years and older will be overweight, including more than 890 million adults with obesity.

**Objective:**

Exercise interventions based on mobile health (mHealth) technology are widely available, but the effectiveness and feasibility of interventions using mHealth apps and exercise watches to improve the physical health of male college students with overweight and obesity are unknown, and this study compares the effects of online interventions carried out by mHealth technology and offline interventions guided by physical trainers on the physical health of male college students with overweight and obesity.

**Methods:**

This study used a randomized controlled trial with a pretest-posttest design, and participants were randomly divided into an online group, an offline group, and a control group. The online group exercised online through the fitness app, and the offline group was instructed by a professional trainer to exercise offline, and both groups wore sports watches to monitor their activities, and the training content was the same. The control group did not carry out any intervention.

**Results:**

At the end of the intervention, the BMI of the online and offline groups decreased by 0.89(SD 1.17) and 0.68(SD 0.94)kg/m², respectively (*P*<.01), and the body fat rate decreased by 1.69%(SD 2.24) in the online group and 2.25% (SD 3.20)in the offline group (*P*<.01). Several physical fitness indicators, such as muscle mass, increased significantly by an average of 1115.23 (SD 1765.42) g in the online group and 1377.74(SD 2203.05)g in the offline group (*P*<.05), and lung capacity improved by 536.82 (SD 745.55) and 450.35 (SD 664.47)ml in the online and offline groups, respectively (*P*<.05). The changes in these indicators between the online and offline groups were not significantly different (*P*>.05). Additionally, a dose-effect relationship was found between the amount of physical activity and the rate of change in body fat, with a 6.9% increase in the rate of change in body fat for every 1 MET*h increase in moderate-intensity physical activity, and a 5.4% increase in the rate of change in body fat for every 1 MET*h increase in high-intensity physical activity.

**Conclusions:**

Exercise intervention based on mHealth technology effectively improves the physical fitness of male college students with overweight and obesity, and the effect is consistent with offline intervention. There was a significant dose-effect relationship between the amount of physical activity and the rate of change in body fat, which provided a scientific basis for the development of personalized training programs.

## Introduction

Obesity has become one of today’s global health challenges. According to the World Health Organization, in 2022, a total of 2.5 billion adults aged 18 years and older will be overweight, including more than 890 million adults with obesity. This equates to 43% of adults aged 18 years and older (43% of males and 44% of females) being overweight [1]. Exercise is the most effective nonpharmacological way to improve obesity, and some studies have shown that exercise can significantly improve the body shape of male college students with obesity [2] and promote physical health [3]. Obesity brings people not only great physical health risks, but also unappreciated psychological harm. Obesity may seriously affect college students’ learning and mental health [1], and these potential risks have been confirmed in studies that obesity is correlated with both self-esteem and academic performance [4], so some studies have referred to the verbal and physical abuse endured by people with overweight as weight stigma and confirmed that weight stigma is negatively correlated with self-esteem in people with overweight [5]. Effective exercise interventions can help people with overweight restore their self-confidence [6], but how to help people with overweight face the enormous psychological pressure and external views of effective exercise interventions is an urgent problem. Offline exercise in an open environment, always surrounded by the outside world’s astonished eyes, part of the population will be under this invisible pressure to gradually give up training. With the development of science and technology, the development of mobile health (mHealth) technology provides new possibilities to deal with this challenge [7]; therefore, it has become imperative to study exercise interventions based on mHealth technology.

mHealth technology refers to the use of mobile devices and related mobile applications to monitor, assess, and improve an individual’s health and health behaviors [8]. Studies have shown that mHealth technology can effectively improve physical activity levels [9] and lifestyles [10], greatly facilitating people’s lives. Current research mainly focuses on online care for special populations and knowledge popularization [11], and the study population focuses on women with shorter intervention cycles [12], and there is a lack of randomized controlled trials in which mHealth technology combined with exercise intervention is used as an effective intervention, which does not prove its effectiveness and restricts its widespread application.

Wearable devices are currently widely used in remote medical monitoring [13], to help doctors understand individual disease trajectories; in the field of exercise intervention mainly to provide real-time physical activity feedback, with the help of wearable devices,, health intervention and timely adjustment of the intervention program can be obtained promptly, to cultivate self-monitoring of physical activity habits of the research participants, to provide a basis for the objective quantitative evaluation of the behavioral patterns of the research participants, and to improve the effectiveness of health intervention for the participants based on traditional health interventions to improve support for individual personalization of participants, to better match research participants’ personal preferences and characteristics, and to provide the possibility for personalized interventions [14]. Such adjustments often take time to provide feedback, and if the dose-effect relationship between physical activity and physical health indicators can be established, the time can be greatly reduced, the training program can be scientifically and effectively formulated, and the training load can be determined before the training starts, to achieve goal-oriented exercise interventions and stimulate a stronger awareness of exercise.

Currently, on the one hand, whether mHealth can achieve the same effect as offline fitness is still worthy of further debate; on the other hand, weight loss is not only related to exercise; related studies believe that weight loss is also related to daily physical activity. Low physical activity patients endure cardiovascular disease and death risk is significantly higher [15], so it is very important to research the specific role that physical activity can play in exercise intervention. In summary, this study explores the relationship between physical health improvement, physical activity, and body fat percentage in college students with overweight and obesity around mHealth technology.

## Methods

### Study Design and Participants

This was a pre- and posttest study using a randomized controlled experimental design with an additional control group. The 12-week intervention was conducted from April to June 2024 at a comprehensive university.

Participants were eligible if they were aged 20‐30 years and had a BMI ≥24. Exclusion criteria included (1) contraindications to exercise, (2) chronic conditions such as cardiovascular disease or diabetes, (3) infectious diseases such as hepatitis B or tuberculosis, and (4) chronic smoking. All participants provided written informed consent before enrollment. This study was approved by the Ethics Committee of the Academic Committee of Capital University of Physical Education and Sports (code: 2024A063, approval date: April 11, 2024) and registered in the Chinese Clinical Trial Registry (ChiCTR2400092057).

Participants were randomly assigned to 1 of 3 groups (online intervention, offline intervention, or control) using a computer-generated simple randomization method. Group assignment and data analysis were conducted by independent researchers blinded to the intervention.

### Description of the Intervention

Participants in the online and offline groups underwent 12 weeks of aerobic combined strength training, with the same frequency of weekly interventions in both groups, 3 times per week [16] for 80 minutes (10 minutes warm-up, 60 minutes training, and 10 minutes relaxation (Multimedia Appendix 1) [17].

The online group was taught how to use the sports watch by the researcher during the baseline test, as well as safety education. After completing the baseline test, online training was organized and supervised through Tencent Conference (Tencent Holdings Limited). Each person conducted 3 exercise interventions per week, independently selected personalized training programs for the training situation, wore the exercise watch during training, and trained independently against the demonstration videos via the First Body Fitness app. At the end of each training session, a training punch card was posted in the WeChat group in the form of a picture or video. Of the online group of participants according to their own training, the day before participating in the training, they chose the training group they needed to practice in. According to the training content, they were divided into 3 groups of A, B, and C (strength training + aerobic training) training content composition, with the 3 A, B, and C groups corresponding to the 3 time periods of all participants for which to practice the content of the same, to facilitate the supervision of the researchers. The order of the 3 weekly training sessions could be chosen freely.

In the offline group, each participant was instructed by a professional physical trainer to perform 3 exercise interventions per week. In the offline group, strength training was performed with dumbbells, plus abdominal curls; aerobic training was performed in the form of running for 30 minutes each time, totaling 1 set. Warm-up and relaxation were performed for 10 minutes before and after the exercises. Three times a week, the training is carried out per the prescribed program and cannot be switched or adjusted.

Online and offline groups are required to wear sports watches to measure physical activity data all day long and upload them to the backstage punch card through the Shouti Fitness app on time every day. The control group was not required.

### Study Measurements

#### Body Composition Test

The body composition test was performed using a dual-energy x-ray bone densitometer. Participants are required to stay properly hydrated the day before and on the day of the test, avoid drinking and eating for 2 hours before the test, and change into light clothes in advance. During the test, shoes and socks are removed, no metal jewelry can be worn, and the participant is required to lie flat on the center line of the test bed area, symmetrically on the left and right sides, with palms up against the sides of the body placing the feet straight, and no talking during the test. The duration of each test was 10‐15 minutes [18].

#### Maximum Oxygen Uptake Test

Participants were asked to start from a stationary state, wearing K5 equipment, using the Bruce test method to: 2.7 km/h speed, 10% gradient; 4 km/h speed, 12% gradient; 5.5 km/h speed, 14% gradient; 6.8 km/h speed, 16% gradient; 8 km/h speed, 18% gradient, adjusting the speed and gradient every 3 minutes, until they could not continue to run, usually ending around 12 minutes [19].

#### Maximum Strength Test

Participants were asked to learn the barbell bench press and hard pull squat movement pattern, starting with an empty bar and gradually increasing the barbell weight. Participants cannot perform strenuous physical activity in the 24 hours before the test. Then on the day of the test in the physical fitness gym, by completing for a number of times the maximum strength of the test movements to complete the preparatory activities, was selected the participant predicted weight to start the test, gradually made incremental, until the inability to complete the prescribed movement. Then, we recorded the last successful 1 repetition maximum, usually obtained within 4 times, with rest 3‐5 minutes between 2 tests [20].

#### Physical Fitness Tests

Lung capacity, standing long jump, 1000 m, 50 m, seated forward bending, and pull-up tests were conducted per the provisions of the National Physical Fitness Standards for Students (revised 2014) [21].

### Statistical Analysis

In this study, we used previous studies [22] to estimate the required sample size. Assuming an effect size of approximately 0.85 (based on changes in body fat percentage), a 1-tailed α level of .05, a statistical power of 0.50, and a prepost correlation coefficient of 0.80, we determined that a minimum of 22 participants per group would be needed. Accounting for a 15% dropout rate, we recruited 24 participants per group to ensure sufficient power.

In this study, the experimental indicators were analyzed using paired tests, ANOVA, and a nonparametric test “Wilcoxon signed rank sum test” was used for some of the data that did not conform to normal distribution.

### Ethical Considerations

The studies involving human participants were reviewed and approved by the Ethics Committee of the Academic Committee of Capital University of Physical Education and Sports and registered in the Chinese Clinical Trial Registry (ChiCTR2400092057). Informed consent was obtained from the participants’ legal guardians or next of kin, following a thorough explanation of the study's nature, objectives, potential risks, and benefits. Participants were explicitly informed of their right to withdraw from the study at any time without facing any penalty. All personal data and identities were kept confidential and protected in accordance with relevant local, national, and international privacy regulations. If applicable, compensation for participation was provided in line with institutional guidelines.

## Results

### Study Sample Characteristics

**Figure 1.**
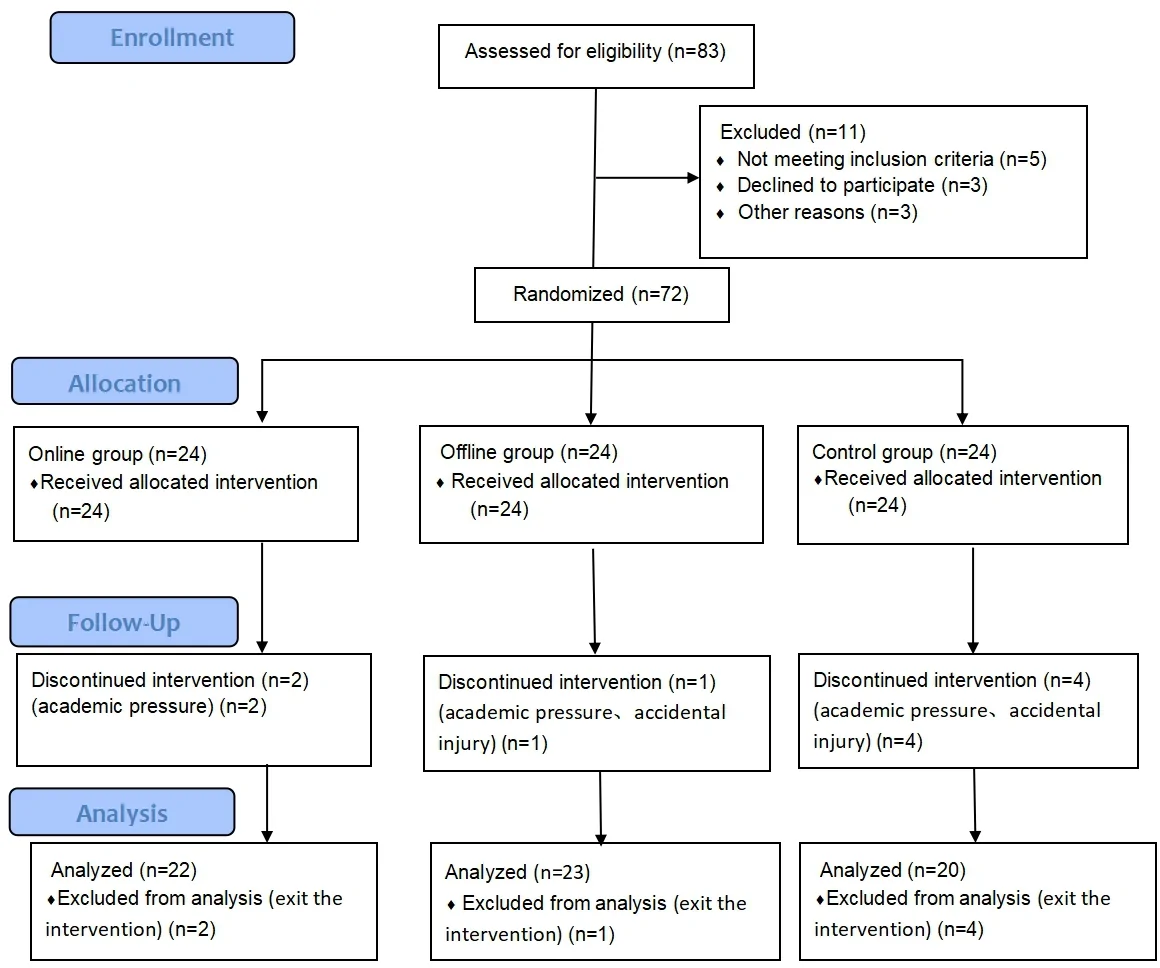
Flowchart of participant screening.

As shown in the figure, 83 participants were recruited for this study, and a total of 72 were enrolled. The other 11 participants were excluded because they did not sign the informed consent form (n=8) and were unwilling to participate (n=3). During the 12-week intervention period, a total of 2 people dropped out of the online group, 2 people dropped out of the offline group, and 4 people dropped out of the control group, with no significant differences in baseline characteristics among the 3 groups (Figure 1 and Table 1).

**Table 1.** Baseline characteristics.

Norm	Online group (n=22), mean (SD)	Offline group (n=23), mean (SD)	Control group (n=20), mean (SD)	*F* test (*df*)	*P* value
Age	23.23 (2.64)	23.57 (2.47)	23.35 (2.66)	0.099 (2)	.91
Height (m)	1.77 (0.05)	1.78 (0.06)	1.77 (0.05)	0.461 (2)	.63
Weight (kg)	89.62 (10.47)	92.14 (8.83)	91.48 (12.01)	0.348 (2)	.71
BMI (kg/m^2^)	28.57 (2.68)	29.05 (2.89)	29.23 (3.11)	0.298 (2)	.74
Body fat percentage (%)	32.07 (5.36)	33.23 (4.32)	33.85 (5.66)	0.660 (2)	.52
Muscle mass (g)	57,180.32 (4685.74)	59,116.57 (5651.81)	56,578.0 (7108.65)	1.127 (2)	.33
Standing long jump (m)	2.22 (0.16)	2.18 (0.16)	2.13 (0.13)	1.852 (2)	.17
1000 m (min)	4.73 (0.86)	4.91 (0.69)	5.00 (0.79)	0.675 (2)	.51
50 m (s)	8.07 (0.69)	8.24 (0.53)	8.34 (0.51)	1.195 (2)	.31
Seated forward bending (cm)	5.04 (8.31)	2.21 (5.92)	1.27 (6.26)	1.720 (2)	.19
Pull-ups (pcs^a^)	1.05 (2.03)	0.39 (0.78)	0.40 (0.88)	1.655 (2)	.20
Bench press 1RM^b^ (kg)	57.16 (13.79)	64.67 (14.07)	61.25 (15.68)	1.515 (2)	.23
Deep squat 1RM (kg)	96.25 (14.88)	98.80 (24.53)	86.88 (20.03)	2.009 (2)	.14
Hard pull 1RM (kg)	96.82 (22.04)	102.39 (19.68)	91.25 (13.27)	1.869 (2)	.16
Lung capacity (ml)	5244.36 (632.86)	5304.26 (925.62)	5367.0 (552.93)	0.148 (2)	.86
Maximum oxygen uptake ml/(kg-min)	47.98 (8.73)	47.81 (5.84)	46.19 (6.68)	0.392 (2)	.68

a pcs: pieces.

b 1RM: 1 repetition maximum.

### Intervention Effects

As can be seen from Table 2, the *P* value of the difference between the pre- and posttest data of each variable in the online group and the offline group is greater than .05, and there is no significant difference.

In the online group and the control group, except for “muscle mass,” the *P* value of the difference between the pre- and posttest data of each variable is less than .05, which can be regarded as a significant difference.

In the offline group and the control group, except for “bench press 1 repetition maximum,” the *P* value of the difference between the pre- and posttest data of each variable is less than .05, which can be regarded as a significant difference.

**Figure 2.**
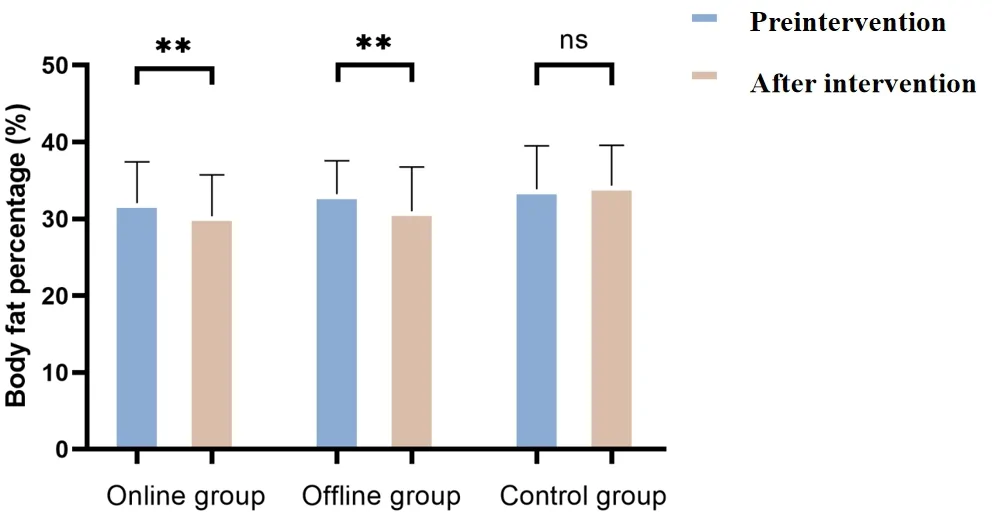
Differences between groups in body fat percentage.

The difference in the change in body fat percentage before and after the experiment among the 3 groups of participants showed a significant difference in the within-group paired-sample *t* test analysis, with the online group (t=3.535,df=21, *P*=.002), the offline group (t=3.373, df=22,*P*=.003), and the control group (t=-1.567,df=19, *P*=.13). In the 1-way ANOVA test of variance between groups, there was a significant difference (*F*_X_=7.364,df=2, *P*=.001), in which there was a significant difference between the online group and the control group (*P*<.01), the offline group and the control group (*P*<.01), and there was no significant difference between the online group and the offline group (Figure 2).

**Table 2.** Comparison between groups. Age, baseline physical characteristics, and any other factors were controlled as covariates.

Norm	Online group (n=22), mean (SD)	Offline group (n=23), mean (SD)	Control group (n=20), mean (SD)	*P* value (online-offline)	*P* value (online-control)	*P* value (offline-control)
BMI (kg/m^2^)	–0.89 (1.17)	–0.68 (0.94)	0.24 (0.78)	.76	.001	.009
Muscle mass (g)	1115.23 (1765.42)	1377.74 (2203.05)	–49.55 (1310.40)	.88	.10	.03
Body fat percentage (%)	–1.69 (2.24)	–2.25 (3.20)	0.48 (1.37)	.72	.001	.007
Lung capacity (ml)	536.82 (745.55)	450.35 (664.47)	3.50 (79.62)	.27	.003	.01
Maximum oxygen uptake ml/(kg-min)	3.72 (5.83)	4.27 (5.67)	–0.46 (1.15)	.92	.01	.001
Standing long jump (m)	0.10 (0.17)	0.07 (0.13)	–0.01 (0.04)	.43	.04	.02
1000 m (min)	0.12 (0.26)	0.11 (0.26)	–0.07 (0.14)	.95	.03	.02
50 m (s)	0.32 (0.56)	0.41 (0.61)	–0.06 (0.16)	.36	.04	.02
Seated forward bending (cm)	3.11 (4.73)	3.86 (5.21)	–0.19 (0.80)	.82	.048	.002
Pull-ups (pcs^a^)	1.36 (1.97)	1.57 (2.23)	0.05 (0.39)	.82	.04	.03
Bench press 1RM^b^ (kg)	7.07 (10.12)	7.39 (13.04)	–0.13 (2.98)	.59	.003	.10
Deep squat 1RM (kg)	9.91 (13.59)	12.28 (19.13)	–0.63 (3.33)	.84	.003	.01
Hard pull 1RM (kg)	11.14 (14.24)	9.67 (18.53)	–0.50 (2.88)	.93	.001	.04

a pcs: pieces.

b 1RM: 1 repetition maximum.

### Changes in Weekly Average Moderate- to High-Intensity Physical Activity

**Figure 3.**
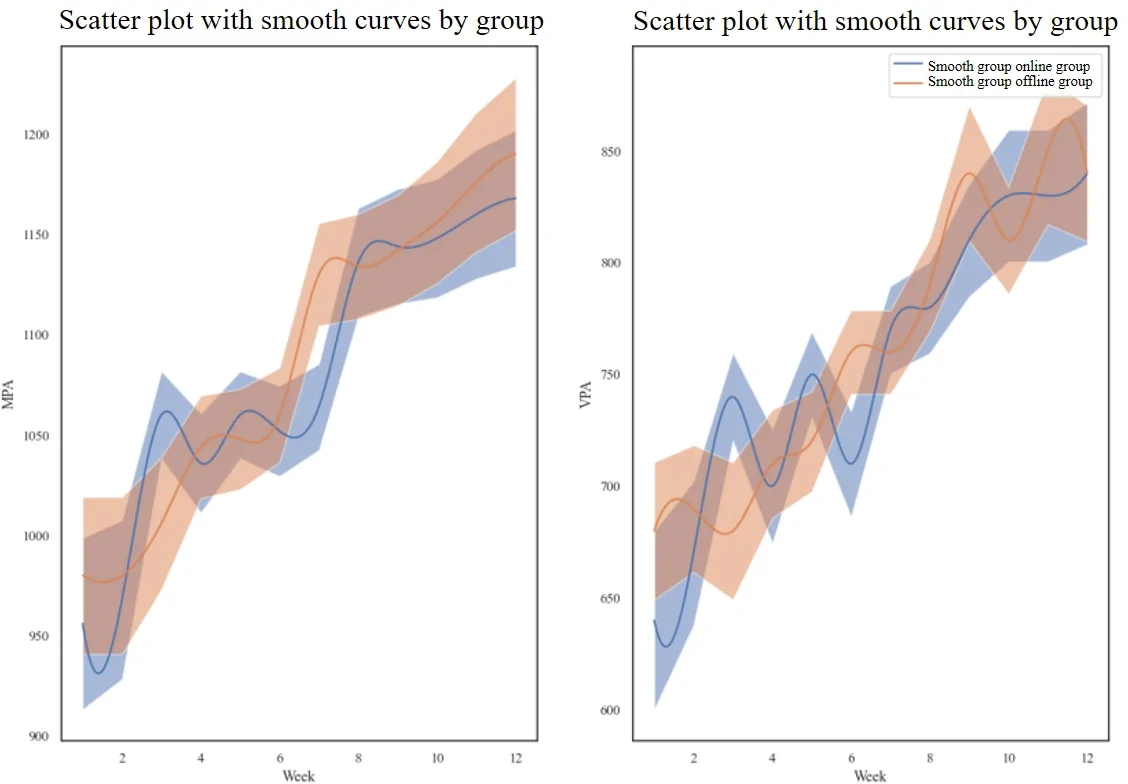
Changes in the amount of moderate to high-intensity physical activity. MPA: moderate physical activity; VPA: vigorous physical activity.

The mean value of weekly moderate-intensity physical activity in the online group was 1087 MET*MIN; in the offline group, it was 1079 MET*MIN. A *t* test for independent samples revealed that there was no significant difference in the amount of moderate-intensity physical activity between the 2 groups. The mean weekly high-intensity physical activity in the online group was 761 MET*MIN, and in the offline group, it was 756 MET*MIN. There was no significant difference in the amount of high-intensity physical activity between the 2 groups as found by independent samples *t* test (Figure 3).

The mean value of total weekly MET*MIN in the online group was 1848 MET*MIN, and the mean value of total weekly MET*MIN in the offline group was 1835 MET*MIN, which far exceeded the World Health Organization recommendation of 900 MET*MIN per week.

**Figure 4.**
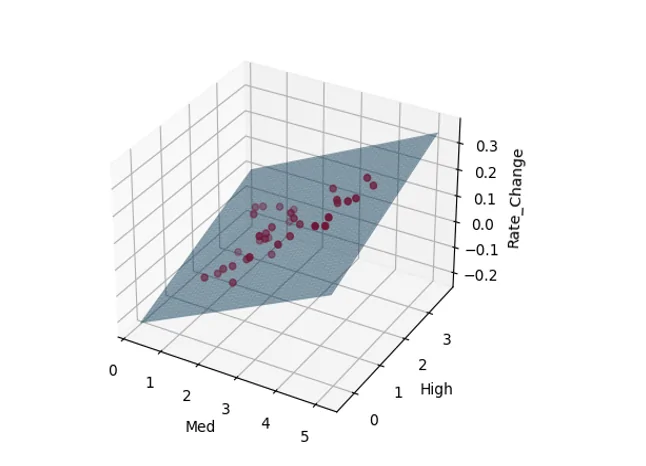
Linear regression model. Med: medium.

Relationship between the amount of physical activity and the rate of change in body fat: Rate_change=–0.2108+0.0690 * med+0.0544 * high. The model is interpreted as follows: all else being equal, each unit increase in moderate-intensity exercise time will increase the rate of change in body fat by 0.0690 units; each unit increase in high-intensity exercise time will increase the rate of change in body fat by 0.0544 units (Figure 4).

The calculated variance inflation factor value for med and high was 1.5915, and there was no significant multicollinearity. Additionally, as the duration of exercise was calculated separately for each intensity, it can be assumed that there is no interaction. Y=–0.2108 + 0.0690 * med+0.0544 * high (). On linear regression (95% CI 0.025‐0.975), the regression coefficient for moderate physical activity was 0.0690 (SE 0.009; 2-tailed *t*_33_=7.644 [model df=2]; *P*=.099; 95% CI 0.051‐0.087), and that for vigorous physical activity was 0.0544 (SE 0.014; 2-tailed *t*_33_=3.968 [model df=2]; *P*=.46; 95% CI 0.026‐0.082).

### Testing

Validation of the test set data yields a mean square error of 0.0021 (Table 3).

**Figure 5.**
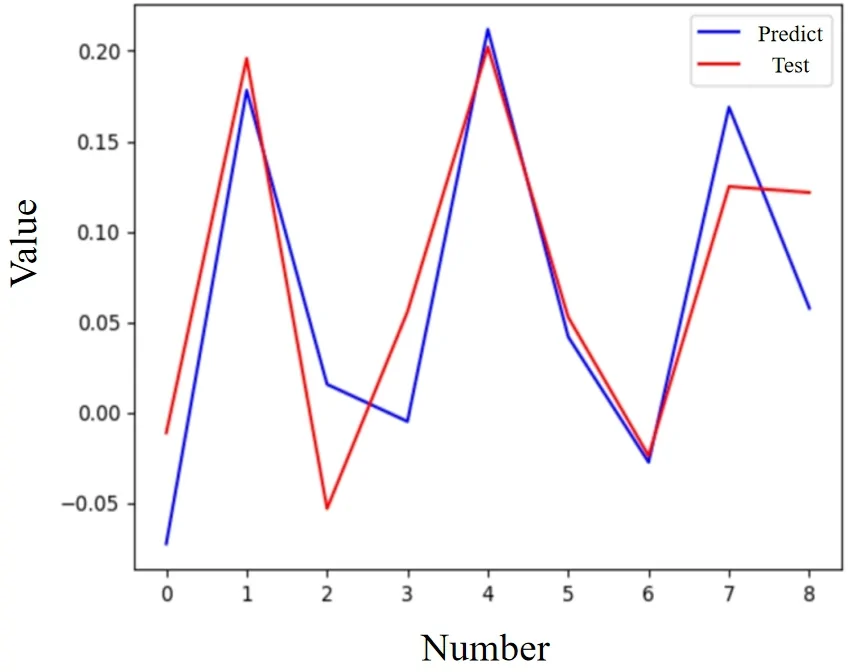
Graph of true values of the test set and training predictions.

In the graph of the true values of the test set and the training predictions, as shown in Figure 5, it can be seen that the overall values are relatively close to each other, and due to the small sample size itself, there will be a certain degree of error.

**Table 3.** Validation of parameters related to linear regression–related.

Validation of linear regression–related parameters	Relationship and value
Regression intercept	W0=−0.21080691390909367
Regression coefficient	W1=(0.06900332-0.05437355)
*R*^2^ coefficient of determination	0.8428
Mean square error	0.0021
Mean absolute value error	0.0378
Median absolute value error	0.0439

## Discussion

### Principal Findings

In this study, the physical health of the online group and the male college students with overweight and obesity guided by professional physical fitness trainers based on mHealth technology interventions was significantly improved, and the amount of physical activity of the online group gradually increased with the interventions, which confirms the hypothesis that the interventions using the mHealth app and sports watch can effectively improve the physical health of the male college students with overweight and obesity; and second, this study found that there is a significant dose-effect relationship between the rate of change of body fat and the amount of physical activity. Second, it was found that there was an obvious dose-effect relationship between the rate of change in body fat and the amount of physical activity, specifically Y=–0.2108+0.0690 * med+0.0544 * high, which provides a reliable theoretical basis for the development of scientific training programs.

### Effectiveness of mHealth Technology–Based Exercise Interventions to Improve Body Fat Percentage

The effectiveness of mHealth technology-based exercise interventions in improving body fat percentage has gained widespread attention. With the proliferation of smartphones and wearable devices, these technologies offer flexible and personalized exercise programs for people with obesity and overweight. Several high-level studies have demonstrated that mHealth interventions are effective in promoting reductions in body fat percentage.

A growing body of research validates the effectiveness of mHealth technology in body fat percentage management. For example, it was found that participants who used an mHealth app reduced their body fat percentage by an average of 1.22% over 12 weeks. This study highlighted the importance of personalized feedback and goal setting, where participants were provided with real-time data through the app, leading to enhanced self-monitoring skills and motivation to exercise [23].

Additionally, Wang et al [24] found that participants’ activity level and exercise intensity significantly increased when wearing a smartwatch for exercise monitoring, which ultimately led to an effective decrease in body fat percentage. The real-time data feedback from wearable devices helped participants to adjust their exercise intensity, which improved the efficiency and effectiveness of exercise.

In this study, after 12 weeks of mHealth technology intervention, it was found that the body fat rate of the online group decreased significantly, which was basically the same as the situation of the online group, so the body fat rate of people with obesity can be effectively improved by mHealth technology.

However, despite numerous studies confirming the effectiveness of mHealth technology, some potential limitations need to be noted. Most of the current studies have small sample sizes and focus on specific populations, and the generalizability of the results needs to be verified. Additionally, individual factors such as motivation, social support, and lifestyle may have a significant impact on the effectiveness of the intervention, and these factors need to be explored in greater depth in future studies.

### Quantitative Effectiveness of Physical Activity and Fat Loss of the Intervention

Body fat is one of the most important indicators of an individual’s health status, and the role of physical activity level in body fat changes has received increasing attention. The amount of physical activity is not only an important factor influencing the change of body fat [25], but it can also be used as an effective reference indicator to predict and intervene in the change of body fat [26].

Studies have shown that there are differences in the effects of different intensities of physical activity on body fat [27]. Both high-intensity interval training and moderate-intensity continuous aerobic exercise have been shown to have significant effects on body fat reduction. These types of exercise not only expend large amounts of energy during exercise, but also further promote fat burning by increasing postexercise oxygen consumption. Therefore, the exercise intensity and type of physical activity are closely related to the rate of change in body fat, further emphasizing its significance as a reference for body fat change. The dose-effect relationship between physical activity and the rate of change in body fat is unclear [28]. This study conducted linear regression analysis around moderate-intensity and high-intensity physical activity and body fat change rate and found that there is a dose-effect relationship between physical activity and body fat change rate, and the current study around children [29], the elderly [30], so it can provide a more scientific weight loss reference for male college students with overweight and obesity, but due to the small sample size, the follow-up can be further improved to improve the dose-effect relationship of the accuracy. The use of physical activity as a reference for the rate of change of body fat can provide personalized health management for different populations. By monitoring the physical activity of individuals, the level of energy consumption can be assessed, thus providing data support for body fat management; second, high body fat is closely related to a variety of chronic diseases [31]. By monitoring and adjusting the amount of physical activity, the level of body fat can be effectively controlled, and the risk of chronic diseases can be reduced. Future research and practice should continue to explore how to more accurately use physical activity as an indicator to optimize health intervention programs and improve the overall effectiveness of body fat management.

### Effectiveness of mHealth Technology Exercise Interventions to Improve Physical Health

With the popularity of smart devices, mHealth technologies are developing rapidly and are widely used around the world. These technologies help users monitor health indicators, record daily activities, get health advice, and conveniently develop personalized exercise plans. Common mHealth technologies include smart wearables, health tracking apps, telemedicine, and health education and motivation platforms. Wearable devices are more common mHealth devices, mainly used for daily monitoring of physical indicators [32], to provide convenience for different groups [33]. At present, the use of wearable devices to help college students improve health behaviors in intervention experiments is still in the exploratory stage, though the study found that the use of wearable devices to monitor daily physical activity is not effective in promoting changes in physical activity [34,35], this result may be related to the lack of personalized feedback after accessing wearable devices [36,37], whereas technologies such as mHealth apps and telemedicine are now able to provide effective assistance to patients in posttreatment rehabilitation [13,38]; therefore, the question of whether combining wearable devices with mHealth apps can provide greater assistance to counterparts has become an emerging one.

In traditional exercise interventions, a uniform training program is usually adopted, and the offline intensive training has psychological and other disturbing factors for people with overweight and obesity [39], which can seriously affect the health promotion effect of people with obesity. Compared with the traditional exercise intervention, this study measured the physical activity and various physical indicators through wearable devices [40], combined with the first body fitness app for male college students with overweight and obesity to push personalized training program feedback, and through social software for goal setting, self-monitoring, and other behavioral interventions [41], so that the training process was scientific, individualized, and achieved a good intervention effect, proving that the effectiveness of mHealth technology for exercise intervention is effective [42]. In terms of body shape, the online intervention group’s body fat rate decreased by an average of 1.69% (*P*<.01); muscle mass increased by an average of 1115.23 g (SD 1765.42 g; *P*<.01), and there was also a significant improvement in physical fitness and physical function. Currently, although many health and fitness apps aim to promote increased physical activity or exercise participation [43], with the primary outcome being changes in body weight [44], few apps aim to improve the quality of functional movement and physical fitness, while meeting individual needs and providing personalized exercise guidance, so this study informs the aspect of mHealth in enhancing physical fitness.

### Study Strengths and Limitations

This study demonstrates notable strengths, including the innovative use of mHealth technology, which enhances participant engagement and allows for real-time monitoring of physical activity, thereby fostering adherence to exercise interventions. The objective data collected from wearable devices, such as fitness trackers, ensures that physical activity measurements are accurate and reliable, minimizing the biases often associated with self-reported data. Additionally, the randomized controlled trial design strengthens the causal relationship between the interventions and improvements in physical health metrics among male college students with overweight and obesity. This robust methodological approach is one of the key strengths of this study, allowing for more confident conclusions regarding the effectiveness of mHealth interventions.

However, several limitations should be acknowledged. First, the relatively small sample size may restrict the generalizability of the findings. Although the sample size was appropriate for the scope of this study, it may not fully represent the wider population of college students with overweight and obesity. Despite this, we believe this study’s randomized design mitigates the impact of this limitation, as it reduces selection bias and increases the internal validity of the results. Additionally, the short duration of the intervention limits the ability to assess the long-term sustainability of health benefits. The 12-week intervention period provided valuable insights into short-term changes in physical health, but further research with longer follow-up durations is necessary to evaluate the lasting effects of such interventions.

Regarding confounding factors, while we controlled for baseline physical fitness and demographic variables (such as age and BMI), there were still unmeasured factors, such as diet, sleep patterns, and genetic predispositions, that could influence the outcomes. We acknowledge that these factors were not fully controlled for in this study. However, we believe that the use of objective activity data, in combination with the randomized controlled design, reduces the impact of potential confounders on the observed effects, providing a more accurate reflection of the intervention’s impact on physical health.

Moreover, the online intervention group was allowed to freely choose the order in which they completed training modules, which may have introduced variability in the intervention delivery and potentially biased the outcomes. We acknowledge this as a limitation and recommend that future studies implement a more standardized training sequence to enhance consistency across participants.

Statistically, potential multicollinearity between different exercise intensities was considered. Despite our best efforts to control for the variables involved, further studies should consider larger sample sizes and longer intervention times to better explore the role of interactions and to ensure that findings can be generalized more widely.

### Conclusions

mHealth-based technology was as effective as offline exercise interventions in improving body fat percentage and athletic quality in college students with obesity. There was a quantitative-effect relationship between the amount of physical activity and the amount of body fat reduction of the exercise intervention, with the rate of change in body fat increasing by 0.0690 and 0.0544 for every 1-megaton increase in moderate-intensity and high-intensity exercise time, respectively.

Online and offline training were basically similar for male college students with overweight and obesity in terms of improvement in body shape, physical fitness, and physical function. Therefore, online training based on mHealth technology is feasible and effective.

The dose-effect relationship between the amount of physical activity and the rate of change in body fat can provide a more scientific basis for training, develop personalized training programs, obtain quantitative feedback on energy expenditure, and improve training efficiency. The online training in this study also introduced interventions such as wearable testing devices on top of mHealth apps, and the effectiveness of mHealth apps as a stand-alone intervention for implementing health behavior interventions can be explored separately in the future to continuously improve the potential functions of mHealth apps.

## Supplementary material

10.2196/69451 Multimedia Appendix 1 Additional tables.

10.2196/69451 Checklist 1 CONSORT-EHEALTH (Consolidated Standards of Reporting Trials of Electronic and Mobile Health Applications and Online Telehealth) checklist.

## Abbreviations

mHealth: mobile health
