# Correction: Effects of an Exercise Intervention Based on mHealth Technology on the Physical Health of Male University Students With Overweight and Obesity: Randomized Controlled Trial

**DOI:** 10.2196/106884

**Published:** 2026-09-03

**Authors:** Qinlong Zhang, Zhen Li, Lei Jiang, Yanan Gao, Tingjun Gong, Jian Li, Zhixiong Zhou

**Affiliations:** 1 Capital University of Physical Education and Sports, No.11, North Third Ring West Road, Haidian District, Beijing, 100191, China, 86 010 82099489; 2 School of Physical Education and Sport Science, Fujian Normal University, Fuzhou, China

In “Effects of an Exercise Intervention Based on mHealth Technology on the Physical Health of Male University Students With Overweight and Obesity: Randomized Controlled Trial” [1], the authors noted the following corrections.

First, the Trial Registration statement in the Abstract has been corrected.

***Original:***

Chinese Clinical Trial Registry ChiCTR2200063892; https://www.chictr.org.cn/showproj.html?proj=180197

***Revised:***

Chinese Clinical Trial Registry ChiCTR2400092057; https://www.chictr.org.cn/showproj.html?proj=248450

Second, in the Methods section under “Study Design and Participants,” the statement on ethical approval and trial registration has been amended to reflect the correct approval date and registration details.

***Original:***

This study was approved by the Ethics Committee of the Capital Institute of Sports (code: 2024 A063, approval date: December 26, 2017) and registered in the Chinese Clinical Trial Registry (ChiCTR2200063892).

***Revised:***

This study was approved by the Ethics Committee of the Academic Committee of Capital University of Physical Education and Sports (code: 2024A063, approval date: April 11, 2024) and registered in the Chinese Clinical Trial Registry (ChiCTR2400092057).

Third, in the Ethical Considerations section, the statement has been updated for consistency.

***Original:***

The studies involving human participants were reviewed and approved by the Ethics Committee of the Capital Institute of Sports and registered in the Chinese Clinical Trial Registry (ChiCTR2200063892).

***Revised:***

The studies involving human participants were reviewed and approved by the Ethics Committee of the Academic Committee of Capital University of Physical Education and Sports and registered in the Chinese Clinical Trial Registry (ChiCTR2400092057).

These corrections are supported by the original ethics approval documentation (Approval No. 2024A063, issued on April 11, 2024, by the Ethics Committee of the Academic Committee of Capital University of Physical Education and Sports) and the corresponding trial registration ChiCTR2400092057.

The correction will appear in the online version of the paper on the JMIR Publications website together with the publication of this correction notice. Because this was made after submission to PubMed, PubMed Central, and other full-text repositories, the corrected article has also been resubmitted to those repositories.
