# Supplementary material for: Effects of an Exercise Intervention Based on mHealth Technology on the Physical Health of Male University Students With Overweight and Obesity: Randomized Controlled Trial
Source: J Med Internet Res. 2025 Jul 31;27:e69451. doi: 10.2196/69451 (PMC12312992; doi:10.2196/69451)
Supplement: Multimedia Appendix 1 — Additional tables. [file jmir-v27-e69451-s001.docx]

**Table S1.** Offline group training content.

| Offline group | | | | | |
| --- | --- | --- | --- | --- | --- |
| First training | | | | | |
| Warm-up section (10 min) | | | | | |
| Neck circle, Shoulder circle, Wrist rotation, Low side lunge, Marching knee hold | | | | | |
| Basic section (60 min) | | | | | |
| Training movement | Training area | Weight | Number of times/time | No. of groups | Intermittence |
| Dumbbell overhead row | Helper | 15RM(65%1RM) | 15times | 3 | 30‐60 s |
| Dumbbell squat | Leg | 15RM(65%1RM) | 15times | 3 | 30‐60 s |
| Dumbbell flat bench press | Chest | 15RM(65%1RM) | 15times | 3 | 30‐60 s |
| Roll-up | Stomach | Own weight | 15times | 3 | 30‐60 s |
| Run | Whole body | VO2max70% | 30min | 1 | 0 |
| Relaxation section (10 min) | | | | | |
| Arm Stretch, Back Stretch, Waist Stretch, Chest Stretch, Leg Stretch | | | | | |
| Second training | | | | | |
| Warm-up section (10 min) | | | | | |
| Neck circle, Shoulder circle, Wrist rotation, Low side lunge, Marching knee hold | | | | | |
| Basic section (60 min) | | | | | |
| Training movement | Training area | Weight | Number of times/time | No. of groups | Intermittence |
| Dumbbell curl | Helper | 15RM(65%1RM) | 15times | 3 | 30‐60 s |
| Dumbbell squat jump | Leg | 15RM(65%1RM) | 15times | 3 | 30‐60 s |
| Dumbbell Incline Chest Clamp | Chest | 15RM(65%1RM) | 15times | 3 | 30‐60 s |
| Roll-up | Stomach | Own weight | 15times | 3 | 30‐60 s |
| Run | Whole body | VO2max70% | 30min | 1 | 0 |
| Relaxation section (10 min) | | | | | |
| Arm Stretch, Back Stretch, Waist Stretch, Chest Stretch, Leg Stretch | | | | | |
| Third training | | | | | |
| Warm-up section (10 min) | | | | | |
| Neck circle, Shoulder circle, Wrist rotation, Low side lunge, Marching knee hold | | | | | |
| Basic section (60 min) | | | | | |
| Training movement | Training area | Weight | Number of times/time | No. of groups | Intermittence |
| Dumbbell pull | Helper | 15RM(65%1RM) | 15times | 3 | 30‐60 s |
| Dumbbell lunge curl | Leg | 15RM(65%1RM) | 15times | 3 | 30‐60 s |
| Dumbbell Squatting Bird | Chest | 15RM(65%1RM) | 15times | 3 | 30‐60 s |
| Roll-up | Stomach | Own weight | 15times | 3 | 30‐60 s |
| Run | Whole body | VO2max70% | 30min | 1 | 0 |
| Relaxation section (10 min) | | | | | |
| Arm Stretch, Back Stretch, Waist Stretch, Chest Stretch, Leg Stretch | | | | | |

**Table S2.** Online group training content.

| Online group | | | | | | |
| --- | --- | --- | --- | --- | --- | --- |
| First training | | | | | | |
| Warm-up section (10 min) | | | | | | |
| Neck circle, Shoulder circle, Wrist rotation, Low side lunge, Marching knee hold | | | | | | |
| Basic section (60 min) | | | | | | |
|  | Training area | Training movement | Weight | Number of times/time | No. of groups | Intermittence |
| A | Leg | Dumbbell squat | 15RM(65%1RM) | 15times | 3 | 30‐60 s |
|  |  | Dumbbell squat jump | 15RM(65%1RM) | 15times | 3 | 30‐60 s |
|  |  | Dumbbell lunge curl | 15RM(65%1RM) | 15times | 3 | 30‐60 s |
|  | Stomach | Roll-up | Own weight | 15times | 3 | 30‐60 s |
|  | Whole body | Run | VO2max70% | 30min | 1 | 0 |
| Relaxation section (10 min) | | | | | | |
| Arm Stretch, Back Stretch, Waist Stretch, Chest Stretch, Leg Stretch | | | | | | |
| Second training | | | | | | |
| Warm-up section (10 min) | | | | | | |
| Neck circle, Shoulder circle, Wrist rotation, Low side lunge, Marching knee hold | | | | | | |
| Basic section (60 min) | | | | | | |
|  | Training area | Training movement | Weight | Number of times/time | No. of groups | Intermittence |
| B | Helper | Dumbbell curl | 15RM(65%1RM) | 15times | 3 | 30‐60 s |
|  |  | Dumbbell pull | 15RM(65%1RM) | 15times | 3 | 30‐60 s |
|  |  | Dumbbell overhead row | 15RM(65%1RM) | 15times | 3 | 30‐60 s |
|  | Stomach | Roll-up | Own weight | 15times | 3 | 30‐60 s |
|  | Whole body | Run | VO2max70% | 30min | 1 | 0 |
| Relaxation section (10 min) | | | | | | |
| Arm Stretch, Back Stretch, Waist Stretch, Chest Stretch, Leg Stretch | | | | | | |
| Third training | | | | | | |
| Warm-up section (10 min) | | | | | | |
| Neck circle, Shoulder circle, Wrist rotation, Low side lunge, Marching knee hold | | | | | | |
| Basic section (60 min) | | | | | | |
|  | Training area | Training movement | Weight | Number of times/time | No. of groups | Intermittence |
| C | Chest | Dumbbell incline chest clamp | 15RM(65%1RM) | 15times | 3 | 30‐60 s |
|  |  | Dumbbell flat bench press | 15RM(65%1RM) | 15times | 3 | 30‐60 s |
|  |  | Dumbbell squatting bird | 15RM(65%1RM) | 15times | 3 | 30‐60 s |
|  | Stomach | Roll-up | Own weight | 15times | 3 | 30‐60 s |
|  | Whole body | Run | VO2max70% | 30min | 1 | 0 |
| Relaxation section (10 min) | | | | | | |
| Arm Stretch, Back Stretch, Waist Stretch, Chest Stretch, Leg Stretch | | | | | | |
